# Normotensive Posterior Reversible Encephalopathy Syndrome of an Unknown Etiology

**DOI:** 10.7759/cureus.83401

**Published:** 2025-05-03

**Authors:** Ravada R Hemanth Sai Sri Harsha, Praveen Bharti, Sandeep Garg, Mahima Mehra, Pujan Acharya

**Affiliations:** 1 General Medicine, Maulana Azad Medical College, New Delhi, IND; 2 Medicine, Maulana Azad Medical College, New Delhi, IND; 3 Medicine, Lok Nayak Hospital, New Delhi, IND

**Keywords:** cerebrovascular disease, demyelinating disorders, encephalopathy, hydrocephalus, neurodegeneration, papilledema, posterior reversible encephalopathy syndrome, primigravida, seizures, vasogenic edema

## Abstract

Posterior reversible encephalopathy syndrome (PRES) is a neurological disorder commonly associated with hypertension, preeclampsia, eclampsia, and renal dysfunction. However, normotensive presentations without an identifiable etiology remain rare, particularly in pregnancy. We report the case of a 25-year-old primigravida at 22 weeks of gestation who presented with new-onset generalized tonic-clonic seizures, altered sensorium, and bilateral papilledema. Despite the absence of hypertension or predisposing obstetric complications, MRI findings revealed characteristic vasogenic edema in the bilateral parieto-occipital lobes, supporting a diagnosis of PRES. Extensive investigations, including infectious, autoimmune, and thrombotic workups, failed to identify an underlying etiology. The patient’s condition deteriorated, ultimately resulting in mortality. This case highlights an atypical, normotensive presentation of PRES in pregnancy without the common precipitating factors. While PRES is classically considered a reversible condition, this case underscores the potential for poor outcomes in the absence of timely intervention. Additionally, it raises the question of whether encephalitis or encephalopathy may act as potential triggers for PRES in patients without traditional risk factors.

## Introduction

Posterior reversible encephalopathy syndrome (PRES) is a neurological disorder characterized by transient symptoms such as acute headache, altered mental status, visual disturbances, and, in severe cases, coma, which was described by Hinchey J, Chaves C, Appignani B, et al in 1996 [1]. Epidemiological studies showed that it is more common in females than in males [2]. There are two main classical theories proposed for the pathogenesis of this syndrome, The first is severe hypertension that leads to disruption of the brain autoregulation system, consequently resulting in endothelial edema or injury and the other one is endothelial dysfunction caused by circulating endogenous or exogenous toxins [3]. Several underlying conditions have been associated with its development, including hypertensive encephalopathy, preeclampsia, eclampsia, hemolysis, elevated liver enzymes and low platelets (HELLP) syndrome, immunosuppressive or cytotoxic drug use, acute or chronic kidney disease, thrombotic microangiopathies like thrombotic thrombocytopenic purpura and hemolytic uremic syndrome, high-dose corticosteroid therapy, liver failure or transplantation, endocrine disorders, electrolyte imbalances such as hypercalcemia or hyperparathyroidism, bone marrow transplantation, extensive blood transfusion, erythropoietin therapy, and porphyria [4]. Magnetic resonance imaging (MRI) of affected individuals typically demonstrates vasogenic edema in the posterior cerebral regions. In most cases, early identification and management of the underlying cause led to significant clinical and radiological improvement [5] most PRES cases present with acute or subacute neurological manifestations, frequently accompanied by seizures [4,5]. Initially, seizures may be focal before progressing to generalized convulsions, occasionally leading to status epilepticus. Patients may also exhibit alterations in consciousness, ranging from drowsiness and confusion to more severe states such as stupor or coma. Additionally, various visual disturbances, including hemianopsia, blurred vision, and cortical blindness, may be observed in some cases [5]. The term reversible was not fully true because some of these cases would have irreversible course and fail to recover. In a study by Ashlami et al., it was found that 2.2% of PRES hospitalizations had death as the outcome. Inpatient mortality differed between sexes and more occurred in females [6].

Here, we present a rare presentation of normotensive PRES with undetermined etiology even after extensive investigation in a young primigravida patient who ultimately had an unfavorable outcome.

## Case presentation

This is a case of a 22-week primigravida presented to emergency with a single undocumented febrile episode one week prior, followed by two generalized tonic-clonic seizures. Postictally, she remained unconscious, persisting for four days. No history of focal neurological deficits, sensory disturbances, ataxia, tremors, incoordination, bladder dysfunction, or abdominal pain was reported. The patient did not report any history of sensory dysfunction, including numbness, paresthesia, or loss of sensation. There were no complaints of imbalance, swaying to one side while walking, or difficulty maintaining posture. Additionally, the patient did not experience any tremors during voluntary movements or while reaching for objects. There was no reported lack of coordination in daily activities or fine motor tasks. Bladder function remained intact, with no history of urinary incontinence, urgency, or retention. Furthermore, the patient did not experience any episodes of abdominal pain or discomfort. 

The patient had a history of seizure disorder for the past four years and had been on antiepileptic medication for three years. However, she discontinued the medication one year prior to the presentation. There was no history of other chronic illnesses, including asthma, chronic obstructive pulmonary disease (COPD), tuberculosis, or any known contact with tuberculosis. Additionally, there was no history of diabetes mellitus, hypertension, or ischemic heart disease. The patient reported normal bowel and bladder habits, with no complaints of incontinence or changes in frequency. Appetite remained adequate, with no significant weight loss reported. There was no known history of occupational exposure to neurotoxic agents. Sleep patterns were unremarkable, and there were no dietary restrictions or food allergies. The patient denied a history of smoking, alcohol consumption, or substance abuse. However, details regarding the specific medication, dosage, and reason for discontinuation were not available. The patient was a primigravida at 22 weeks of gestation. There were no reported complications during the pregnancy up to the time of presentation. On examination, the patient was hemodynamically stable, with a blood pressure of 110/60 mmHg and a pulse rate of 110 beats per minute. Oxygen saturation was 99% on room air, and capillary blood glucose was 109 mg/dL. There were no signs of pallor, icterus, cyanosis, clubbing, lymphadenopathy, or edema. 

On neurological evaluation, the patient had a Glasgow Coma Scale (GCS) score of E2 V2 M4, indicating a moderate level of consciousness impairment. Bilateral pupils were mid-dilated and reactive to light, with no gaze deviation or abnormal eye movements. A neck examination revealed terminal rigidity, suggesting possible meningeal irritation. Deep tendon reflexes were normal, while the bilateral plantar response was extensor (Babinski sign positive), indicating an upper motor neuron lesion. Respiratory system examination showed equal bilateral air entry, with no added sounds such as wheezing or crepitations. Cardiovascular system assessment was unremarkable, with normal heart sounds and no audible murmurs. Abdominal examination revealed a soft, non-tender abdomen with no evidence of hepatosplenomegaly, palpable masses, or free fluid. In view of the condition, the patient was subjected to all relevant biochemical, hematological, and radiological investigations as shown in Table 1. The laboratory investigations revealed thrombocytopenia with a platelet count of 80,000/µL(low). The rest of the parameters, including hemoglobin, total leukocyte count, absolute eosinophil count, blood urea, serum creatinine, potassium, pH, partial pressure of carbon dioxide (PCO₂), and bicarbonate levels, were within the normal range. 

**Table 1:** It shows important hematological and biochemical investigations done initially. Image credits: Dr. Mahima Mehra, Postgraduate Resident, Department of Medicine, Maulana Azad Medical College. LDH: Lactate dehydrogenase, ALT: Aspartate transaminase, ALT: Alanine transaminase, ALP: Alkaline phosphatase.

Investigation	On day 1	On day 3	Reference range
Hemoglobin	10.5	11.9	Male: 13.8-17.2g/dL, female: 12.1-15.1g/dl
Total counts	7,900	6,800	4000-11000cells/micro-L
Platelets	80,000	1.09 lakh	150000-450000cells/ micro-L
Urea	34	24	Urea: 7-20mg/dL
Creatinine	0.8	0.7	Creatinine: 0.6-1.2 mg/dL
LDH	342	236	LDH; 140-280 U/L
Uric acid	3.8	3.5	Uric acid males: 3.4-7.0 mg/dL, female: 2.4-6.0 mg/dL
AST	103	112	10-40 U/L
ALT	98	94	7-56 U/L
ALP	178	205	44-147 U/L
Total bilirubin	0.7	0.8	0.1-1.2 mg/dL

Chest X-ray showed normal bilateral lung fields, clear costophrenic angles, and no evidence of cardiomegaly. Urine routine microscopy revealed acidic pH, the absence of sugar, and the presence of trace protein. Microscopic examination showed 10 pus cells per high-power field, with no red blood cells detected. Ultrasonography of the abdomen demonstrated a normal liver and spleen in terms of size and architecture, with no evidence of free fluid or lymphadenopathy. Both kidneys were of normal size, and corticomedullary differentiation was well maintained cerebrospinal fluid (CSF) analysis revealed a total cell count of six, all of which were mononuclear. CSF sugar was 78 mg/dL, while protein levels were elevated at 50 mg/dL. The adenosine deaminase (ADA) level was four. Fundoscopic examination showed the presence of bilateral papilledema, suggesting increased intracranial pressure. The initial electroencephalogram showed a background pattern consisting of 3.5-4 Hz bilateral symmetrical posterior predominant delta activity, along with diffuse bilateral background slowing. These findings suggest generalized cerebral dysfunction, which could be indicative of an underlying encephalopathic process. The differential diagnosis includes infective causes such as viral encephalitis, meningoencephalitis, or cerebral malaria. Obstetric-related conditions such as eclampsia or atypical preeclampsia also need to be considered, despite the absence of hypertension or proteinuria. Additionally, the possibility of cortical venous thrombosis should be explored, given the neurological manifestations.

The infectious disease workup showed negative results for malaria (confirmed on repeat testing), dengue (NS1 antigen), leptospirosis, scrub typhus, and viral hepatitis (A, B, C, and E). HIV screening was also negative. Coagulation parameters were within normal limits, with a prothrombin time of 12.7 seconds and an INR of 1.09. However, D-dimer levels were elevated at 1420, which may indicate a prothrombotic state. Serum ammonia was within the normal range at 23, and serum ceruloplasmin levels were also normal. The peripheral smear showed normocytic normochromic red blood cells with a few microcytic hypochromic cells and mild Aniso poikilocytosis. There was no evidence of atypical cells or schistocytes, ruling out microangiopathic hemolytic anemia. The autoimmune and thrombophilia workup was negative. Special investigations like (Table 2) antinuclear antibody (ANA) and antineutrophil cytoplasmic antibody (ANCA) profiles were negative, and antiphospholipid and anticardiolipin antibodies were absent, reducing the likelihood of an autoimmune or antiphospholipid syndrome-related pathology. Complement levels (C3 and C4) were within normal limits, and protein C and protein S levels were also normal, suggesting no significant coagulation dysfunction. Serum homocysteine levels were within the normal range, ruling out hyperhomocysteinemia as a contributing factor. Her CSF autoimmune profile and viral, bacterial, and infectious profile were normal. She was intubated in view of recurrent seizures (Table 2).

**Table 2:** It shows important investigations done to determine etiology. Image credits: Dr. Pujan Acharya, Dr. Mahima Mehra, Postgraduate Residents, Department of Medicine, Maulana Azad Medical College CSF: Cerebrospinal fluid, ANA: antinuclear antibody, ANCA: Antineutrophil cytoplasmic antibody

Test	Result
ANA profile	negative
ANCA profile	negative
Antiphopshpholipid antibodies and anticardiolipin antibodies	negative
C3 and C4 levels	With in normal limits
Protein C and S	With in normal limits
Serum homocysteine levels	With in normal limits
CSF cytology	4 cells all mononuclear
CSF sugar, protein	78mg/dL and 50mg/dL
CSF cultures for bacteria and fungus	negative
CSF CBNAAT and BIOFIRE	negative
Urine routine microscopy	No abnormality detected
CSF for Anti-NMDA-R AMPA GLU – R1, LGI – 1 AMPA GLU – R2, GABA- B	negative
Peripheral smear for malarial parasite	negative
NS1 antigen	negative
Leptospira, scrub typhus and Japanese encephalitis serology	negative

**Figure 1:**
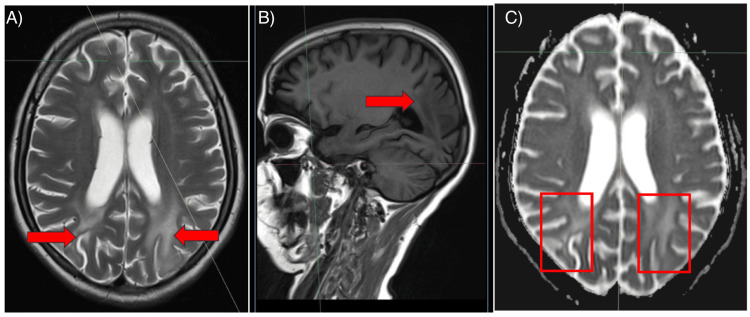
The given MRI image consists of three different sequences commonly used in neuroimaging: Image a) (T2-weighted MRI) – This image shows hyperintense (bright) areas in the parieto-occipital white matter (marked by arrows), which is characteristic of posterior reversible encephalopathy syndrome (PRES). Image b) (T1-weighted MRI) – This sagittal T1-weighted. Image c) (Diffusion-Weighted Imaging [DWI]) – This image shows restricted diffusion in the parieto-occipital regions (highlighted in red boxes). Images taken with permission and written consent of patient relatives. Image credits: Dr. Mahima Mehra, Postgraduate Resident, Department of Medicine, Maulana Azad Medical College.

**Figure 2:**
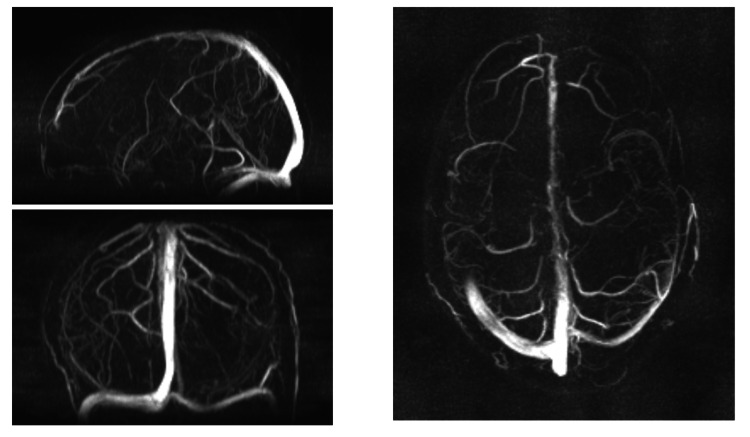
This image shows films of MR venography and these are entirely normal. This image obtained after taking informed and written consent from patient relatives. Image credits: Dr. Pujan Acharya, Postgraduate Resident, Department of Medicine, Maulana Azad Medical College.

As shown in Figure 1, MRI brain findings revealed areas of altered signal intensity involving the subcortical and periventricular white matter of the bilateral parieto-occipital lobes, left temporal lobe, and bilateral middle cerebellar peduncles. Additionally, there were areas of diffusion restriction in the left parietal lobe and bilateral cerebellar peduncles. These findings are suggestive of posterior reversible encephalopathy syndrome (PRES), warranting further evaluation and follow-up. MR venography (Figure 2) showed a normal study, effectively ruling out cortical venous thrombosis as a potential cause of the patient's neurological symptoms. A review after two weeks was recommended for reassessment. The patient was admitted to the intensive care unit (ICU) for close monitoring of blood pressure, neurological status, and renal function. She was placed on a fluid balance chart to monitor for signs of renal deterioration. Given the occurrence of generalized tonic-clonic seizures, the patient was started on IV levetiracetam and midazolam infusion. Given the association between PRES and immune-mediated conditions, a decision was made to administer a course of IV methylprednisolone to reduce inflammation and improve blood-brain barrier integrity, although this is not universally required.

Even after our sincere efforts, the patient's status continued to decline. The patient developed ventilator-associated pneumonia, leading to sepsis and subsequent complications. Despite medical interventions, the condition deteriorated, ultimately resulting in the patient's demise due to these complications.

## Discussion

This case was selected due to its unique presentation of posterior reversible encephalopathy syndrome (PRES) in pregnancy without a single episode of elevated blood pressure or the common precipitating factors typically associated with PRES. In most cases, PRES in pregnancy is linked to conditions such as hypertension, preeclampsia, eclampsia, autoimmune disorders, and renal diseases [7]. However, our patient developed PRES without these established risk factors, raising important clinical questions. Additionally, it raises concerns about the possibility of irreversible cases of PRES, despite its classification as a reversible condition [8]. Another key aspect to explore is whether encephalitis or encephalopathy can act as potential triggers for the development of PRES [8]. Understanding such atypical presentations is crucial for improving early diagnosis and management strategies. This case underscores the need for further research into the underlying mechanisms and risk factors of PRES beyond the traditionally recognized causes, particularly in pregnant patients. 

Interestingly, there is no documented evidence in the literature establishing a direct link between encephalitis or encephalopathy as a trigger for PRES at this age group except in a case report published where a three-year-old infant with influenza-related encephalitis presented with PRES-like features [8]. This raises an important question regarding potential unidentified mechanisms contributing to PRES in patients without traditional risk factors. In severe cases, PRES can lead to fatal outcomes due to progressive cerebral edema, intracranial hemorrhage, and other related complications [9]. The absence of known precipitating factors in this case highlights the need for further research to explore the pathophysiology of atypical presentations of PRES and improve early recognition and management strategies. Although the MRI findings suggested the possibility of posterior reversible encephalopathy syndrome (PRES), it was essential to differentiate it from other conditions (Table 3) with similar clinic-radiological presentations. Several differential diagnoses were considered in this patient, including toxic leukoencephalopathy, acute disseminated encephalomyelitis (ADEM), reversible cerebral vasoconstriction syndrome (RCVS), and progressive multifocal leukoencephalopathy (PMLE) [10]. Toxic leukoencephalopathy is typically associated with exposure to toxins, chemotherapy, or drug abuse, presenting with diffuse white matter changes on imaging [11]. ADEM, an inflammatory demyelinating disorder, can mimic PRES but usually follows an infectious or autoimmune trigger and is characterized by more extensive white matter involvement [12]. RCVS, commonly seen in postpartum females and those with vasoactive drug exposure, presents with similar imaging findings but is distinguished by segmental vasoconstriction on angiography [13]. PMLE, caused by JC virus reactivation in immunocompromised patients, was also considered, but the absence of immunosuppressive conditions made it unlikely [14]. By systematically evaluating these differential diagnoses, PRES remained the most plausible explanation for the patient’s condition. However, the absence of traditional precipitating factors in this case further emphasizes the need for a deeper understanding of atypical presentations of PRES.

**Table 3:** Table differentiating various clinic-radiological syndromes presenting like PRES. Table credits: This table was made by author himself to differentiate the features of this case with other presentations PMLE: Progressive multifocal leukoencephalopathy, RCVS: Reversible cerebral vasoconstriction syndrome, ADEM: Acute disseminated encephalomyelitis, PRES: Posterior reversible encephalopathy syndrome, HIV: Human immunodeficiency virus, MRI: Magnetic resonance imaging

Features in our patient	Features s/o PMLE
Bilateral symmetrical lesions	Relatively assymetrical lesions
Immunocompetent and negative for HIV	HIV positive / immunodeficient
No drug history	
Features in our patient	Features S/o RCVS
No vasospasm on MRI	Vasospasm
No focal deficit and evidence of segmental ischemia	Focal deficit and ischemia present
Features in our patient	Features S/o ADEM
No antecedent event	Antecedent event like vaccination
No focal deficit, no meningism	Meningism and focal deficits
Lesions predominantly in parietal occipital region	Diffuse bilateral symmetrical lesions

## Conclusions

In conclusion, this case highlights a rare presentation of PRES in pregnancy without hypertension or other common precipitating factors. The absence of traditional risk factors raises important questions about alternative mechanisms contributing to PRES. While encephalitis or encephalopathy as potential triggers for PRES remains uncertain, this case underscores the need for further research on atypical presentations. The fatal outcome due to complications such as cerebral edema and sepsis emphasizes the importance of early recognition and management. Differentiating PRES from similar conditions like ADEM, RCVS, and toxic leukoencephalopathy is crucial for accurate diagnosis. Further studies are necessary to explore the pathophysiology of normotensive PRES and improve clinical outcomes. 
